# Whole-brain Functional Connectivity Correlates of Brain Structural Aging in Adult Schizophrenia Patients Compared to Healthy Controls

**DOI:** 10.1192/j.eurpsy.2024.1302

**Published:** 2024-08-27

**Authors:** Y. Panikratova, A. Tomyshev, E. Abdullina, A. Dudina, V. Kaleda, V. Strelets, I. Lebedeva

**Affiliations:** ^1^Laboratory of Neuroimaging and Multimodal Analysis; ^2^Department of Youth Psychiatry, Mental Health Research Center; ^3^Laboratory of Human Higher Nervous Activity, Institute of Higher Nervous Activity and Neurophysiology of RAS, Moscow, Russian Federation

## Abstract

**Introduction:**

Age-related changes of brain functional connectivity, in contrast to brain structure, are understudied in schizophrenia. Importantly, patients with schizophrenia demonstrate an increased difference between the brain-predicted age and chronological age indicating that brain structural aging may be accelerated in this mental disorder (Constantinides et al. Mol Psychiatry 2023; 28 1201-1209). Research on functional connectivity correlates of this process seems to be fruitful.

**Objectives:**

We aimed to search for the brain regions whose resting-state whole-brain functional connectivity is differently associated with brain-predicted age in schizophrenia patients compared to healthy controls.

**Methods:**

Eighty-three male patients with schizophrenia (age range 17.3 – 52.3; mean age 32.1 ± 10.5) and eighty-seven male healthy individuals (age range 18.3 – 53.6; mean age 31.7 ± 10.0) underwent structural MRI and resting-state fMRI (Philips Ingenia 3T scanner). Brain-predicted age was individually estimated using a model trained on independent data based on 68 measures of cortical thickness and surface area, 7 subcortical volumes, lateral ventricular volumes, and total intracranial volume, all derived from T1-weighted MRI scans (Han et al. Mol Psychiatry 2021; 26 5124-39). The associations between the brain-predicted age and the whole-brain global correlation (GCOR) were compared between groups; *t*-contrasts were calculated (one-way ANCOVA covariate interaction via CONN; RRID:SCR_009550; www.nitrc.org/projects/conn). The chronological age was a covariate of no interest.

**Results:**

Schizophrenia patients had a higher difference between brain-predicted age and chronological age (*T*(168) = 2.1; *p* = .036; Cohen’s *d* = 0.32; 95% CI 0.02-0.63). Greater brain-predicted age in schizophrenia patients, compared to controls, was associated with lower functional connectivity of a region in the right Heschl’s gyrus, planum temporale, as well as central opercular and insular cortex with the rest of the brain (*p* < .001 voxelwise, *p*[FDR] < .05 clusterwise; Figure 1).

**Image:
**

**Figure:**
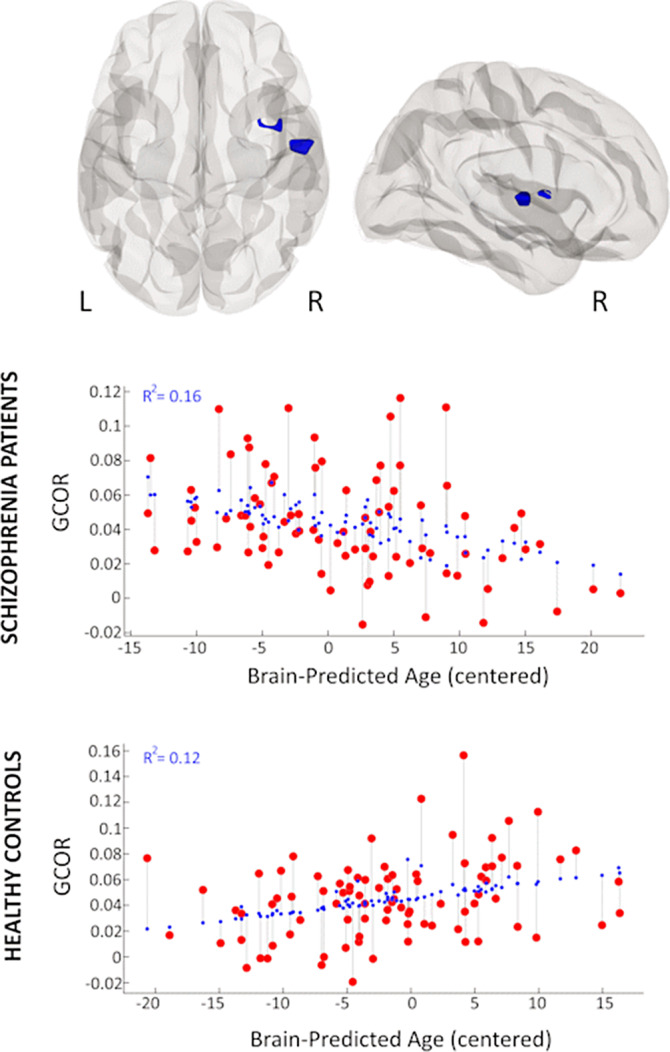

**Conclusions:**

Our results coincide with earlier findings on accelerated brain structural aging in schizophrenia. To the best of our knowledge, the present study is the first to indicate that this process is coupled with a decline of the whole-brain functional connectivity of a region located in the right temporal, insular, and parietal cortices, and this effect is not driven by chronological age. Further studies are needed to clarify the clinical and cognitive correlates of this decline of functional connectivity.

**The study was supported by RSF grant project 22-25-00706.**

**Disclosure of Interest:**

None Declared

